# iNKT Cell Activation Exacerbates the Development of Huntington's Disease in R6/2 Transgenic Mice

**DOI:** 10.1155/2019/3540974

**Published:** 2019-01-15

**Authors:** Hyun Jung Park, Sung Won Lee, Wooseok Im, Manho Kim, Luc Van Kaer, Seokmann Hong

**Affiliations:** ^1^Department of Integrative Bioscience and Biotechnology, Institute of Anticancer Medicine Development, Sejong University, Seoul 05006, Republic of Korea; ^2^Department of Neurology, Seoul National University Hospital, Seoul 03080, Republic of Korea; ^3^Neuroscience and Protein Metabolism Research Institute, Medical College, Seoul National University, Seoul 03080, Republic of Korea; ^4^Department of Pathology, Microbiology and Immunology, Vanderbilt University School of Medicine, Nashville, TN 37232, USA

## Abstract

Huntington's disease (HD) is an inherited neurodegenerative disorder which is caused by a mutation of the huntingtin (HTT) gene. Although the pathogenesis of HD has been associated with inflammatory responses, if and how the immune system contributes to the onset of HD is largely unknown. Invariant natural killer T (iNKT) cells are a group of innate-like regulatory T lymphocytes that can rapidly produce various cytokines such as IFN*γ* and IL4 upon stimulation with the glycolipid *α*-galactosylceramide (*α*-GalCer). By employing both R6/2 Tg mice (murine HD model) and J*α*18 KO mice (deficient in iNKT cells), we investigated whether alterations of iNKT cells affect the development of HD in R6/2 Tg mice. We found that J*α*18 KO R6/2 Tg mice showed disease progression comparable to R6/2 Tg mice, indicating that the absence of iNKT cells did not have any significant effects on HD development. However, repeated activation of iNKT cells with *α*-GalCer facilitated HD progression in R6/2 Tg mice, and this was associated with increased infiltration of iNKT cells in the brain. Taken together, our results demonstrate that repeated *α*-GalCer treatment of R6/2 Tg mice accelerates HD progression, suggesting that immune activation can affect the severity of HD pathogenesis.

## 1. Introduction

Huntington's disease (HD) is an inherited brain disorder which is caused by a mutated form of the huntingtin gene [1]. R6/2 mice expressing a transgenic mutant HTT gene (hereafter R6/2 Tg mice) have been widely used as an HD mouse model. These mice display a progressive neurological phenotype of HD such as tremors, involuntary movements, and seizures [2]. HD pathogenesis is associated with a variety of factors. For example, glutamate uptake is reduced in the prefrontal cortex of HD patients, and an increase in glutamate uptake by upregulation of the glutamate transporter 1 (GLT-1) protects against the development of HD in R6/2 Tg mice [3]. Furthermore, neuronal dysfunction and death are caused by mitochondrial dysfunction and increased oxidative damage [4]. Additionally, several studies have revealed that an increase in pro-inflammatory cytokines such as IL6 and IL8 in plasma correlates with HD disease severity [5, 6]. However, R6/2 Tg mice, when infected with *Toxoplasma gondii*, exhibit decreased CD8^+^ T cell proliferation in the brain and spleen than their noninfected controls do, resulting in premature death [7]. Moreover, mutant HTT is intrinsically expressed in peripheral myeloid cells (e.g., monocyte, macrophages, and microglia) from HD patients, which correlates with impaired migratory functions of these cells [8]. These studies strongly indicate that functional alterations in immune cells that cause immune suppression are linked with the development of HD.

Invariant natural killer T (iNKT) cells express a restricted TCR*α* repertoire (V*α*14-J*α*18 in mice and V*α*24-J*α*18 in humans) and recognize endogenous (e.g., iGb3) and exogenous lipid antigens (e.g., *α*-galactosylceramide (*α*-GalCer) and OCH) presented by the major histocompatibility complex (MHC) class I-like molecule CD1d [9]. Upon activation with *α*-GalCer, iNKT cells secrete a variety of cytokines including both Th1 (e.g., TNF*α*, IFN*γ*, and IL2) and Th2 (e.g., IL4, IL5, IL10, and IL13) cytokines and play either protective or pathogenic roles in autoimmune and allergic disorders [9–11]. In addition, iNKT cells have been implicated in neurodegenerative disorders. In particular, NKT cells are significantly increased in patients suffering from amyotrophic lateral sclerosis (ALS), a fatal neurodegenerative disorder [12], and exhibit harmful effects in an ALS mouse model [13]. Moreover, iNKT cells play protective roles in experimental allergic encephalomyelitis (EAE) in mice, a model for human multiple sclerosis. However, the role of iNKT cells in HD pathogenesis has not been previously explored.

Here, we found that the onset of HD in R6/2 Tg mice is associated with reduced levels of iNKT cells, suggesting the possible involvement of iNKT cells in HD pathogenesis. However, iNKT cell deficiency did not affect HD progression in J*α*18 KO R6/2 Tg mice, indicating that reduction (or lack) of iNKT cells is not a key factor in HD pathogenesis. Interestingly, however, repeated activation of iNKT cells with *α*-GalCer significantly exacerbated clinical symptoms of HD in R6/2 Tg mice. Furthermore, such treatment resulted in increased infiltration of iNKT cells into the brain, which might be related to disease acceleration. Therefore, our results provide evidence that iNKT cells might be one of the critically important immune components in determining disease progression of HD.

## 2. Materials and Methods

### 2.1. Mice

HD Tg mice of the R6/2 line (B6CBA-Tg(HDexon1)62Gpb/3J; hereafter R6/2 Tg mice) were purchased from the Jackson Laboratory. J*α*18 KO mice were kindly provided by Dr. M. Taniguchi (RIKEN, Yokohama, Japan). R6/2 Tg mice were further crossed with J*α*18 KO mice to obtain J*α*18 KO/R6/2 Tg mice. All mice in the present study were maintained at Sejong University and used for experiments at 6-12 weeks of age. The mice were maintained on a 12-hour light/12-hour dark cycle in a temperature-controlled barrier facility with free access to food and water. The mice were fed a *γ*-irradiated sterile diet and provided autoclaved tap water. Age- and sex-matched R6/2 Tg and WT littermate control mice were used for all experiments. The animal experiments were approved by the Institutional Animal Care and Use Committee at Sejong University (SJ-20160704). The present experiments were conducted in a blinded and randomized study.

### 2.2. Intraperitoneal Injection of *α*-GalCer into Mice

Alpha-GalCer was purchased from Enzo Life Sciences Inc. (Farmingdale, NY, USA). R6/2 Tg mice were intraperitoneally (i.p.) injected with *α*-GalCer (2 *μ*g) dissolved in PBS once per week from 5-9 weeks of age. Littermate R6/2 Tg mice injected with PBS were used as negative controls. These mice were also weighed and tested for hindlimb clasping behavior weekly.

### 2.3. Genotyping of Mice

To verify the presence of HTT mutant transgene, genomic DNA from tail biopsies was used to amplify a 170 bp fragment that was only detectable in R6/2 Tg mice carrying the HTT transgene. The following primers were used for genotyping R6/2 Tg mice by PCR: forward 5′-CCG CTC AGG TTC TGC TTT TA-3′ and reverse 5′-TGG AAG GAC TTG AGG GAC TC-3′.

### 2.4. Preparation of Mouse Brain and Clasping Test

The mice were anesthetized with ketamine and xylazine (40 mg/kg and 4 mg/kg, respectively) and subsequently perfused through the left heart ventricle with cold phosphate-buffered saline (PBS) for 3 min to remove cells from the blood vessels. After perfusion, the brain was prepared. For measuring clasping time, 4- or 12-week-old mice were suspended by the tail for 30 s, and the foot-clasping time was measured as follows: 0, 0 s; 1, 0–5 s; 2, 5–10 s; and 3, >10 s [14].

### 2.5. Isolation of Brain-Infiltrating Leukocytes

The brain was sliced into pieces with a scalpel and scissors and subsequently digested with 2.5 mg/mL DNase I (Promega, Madison, USA) and 1 mg/mL collagenase type IV (Sigma, St. Louis, MO, USA) at 37°C in an incubator for 15 min. After the incubation, the digested tissues were dissociated to generate single-cell suspensions using C tubes and a gentleMACS dissociator (Miltenyi, Germany). Subsequently, the cells were filtered through a 70 *μ*m pore size cell strainer and then washed once with PBS containing 10% FBS. The total cell suspension was loaded on a 40%/70% gradient of Percoll (GE Healthcare, Piscataway, NJ, USA). After centrifugation, mononuclear cells (MNCs) were carefully collected from the interphase between the 40% and 70% Percoll layers. Prior to staining, MNCs were washed with PBS and then counted by use of 0.4% trypan blue solution (Welgene, Seoul, Korea) and a hemocytometer.

### 2.6. CD1d/*α*-GalCer Dimer Staining for iNKT Cells

To stain iNKT cells specifically, mCD1d/Ig fusion proteins (CD1d dimer; mouse CD1d DimerX, BD Biosciences, San Jose, CA) were incubated overnight at 37°C with a 40-fold molar excess of *α*-GalCer (in PBS containing 0.5% Tween 20). The staining cocktail was prepared by mixing *α*-GalCer-loaded mCD1d/Ig proteins with FITC- or APC-conjugated anti-mouse IgG1 Ab (clone A85-1, BD Biosciences) at a 1 : 2 ratio of dimer to anti-mouse IgG1 Ab. Subsequently, the mixture was incubated for 2 hr at room temperature.

### 2.7. Cell Isolation and Culture

A single-cell suspension of splenocytes was prepared and resuspended in RPMI complete medium consisting of RPMI 1640 (Gibco BRL, USA) medium supplemented with 10% FBS, 10 mM HEPES, 2 mM L-glutamine, 100 units/mL penicillin-streptomycin, and 5 mM 2-mercaptoethanol. Red blood cells were removed by adding ACK lysis buffer (0.15 M NH_4_Cl, 10 mM KHCO_3_, and 2 mM EDTA in distilled water), and remaining cells were washed with PBS.

### 2.8. Flow Cytometry

The following monoclonal antibodies (mAbs) from BD Biosciences were used: fluorescein isothiocyanate- (FITC-) or phycoerythrin- (PE-) Cy7-conjugated anti-CD4 (clone RM4-5); FITC-, PE-Cy7-, or allophycocyanin- (APC-) conjugated anti-CD3*ε* (clone 145-2C11); PE-Cy7-conjugated anti-CD69 (clone H1.2F3); APC- or biotin-conjugated anti-CD45 (clone PC61); FITC- or PE-conjugated anti-IgG1 (isotype control) (clone R3-34). To perform surface staining, cells were harvested and washed twice with cold 0.5% BSA-containing PBS (FACS buffer). To block Fc receptors, the cells were incubated with anti-CD16/CD32 mAbs on ice for 10 min and subsequently stained with fluorescence-labeled mAbs. Flow cytometric data were acquired using a FACSCalibur flow cytometer (Becton Dickson, San Jose, CA, USA) and analyzed using FlowJo software (Tree Star Inc., Ashland, OR, USA).

### 2.9. Statistical Analysis

Statistical significance was determined using Excel (Microsoft, USA). Student's *t*-test was performed for the comparison of two groups. ^∗^*P* < 0.05, ^∗∗^*P* < 0.01, and ^∗∗∗^*P* < 0.001 were considered to be significant in the Student's *t*-test. Two-way ANOVA analysis was carried out using the VassarStats (http://vassarstats.net/anova2u.html). ^#^*P* < 0.05, ^##^*P* < 0.01, and ^###^*P* < 0.001 were considered to be significant in the two-way ANOVA.

## 3. Results

### 3.1. R6/2 Tg Mice Show Reduced Levels of iNKT Cells at the Onset of HD

**Figure 1:**
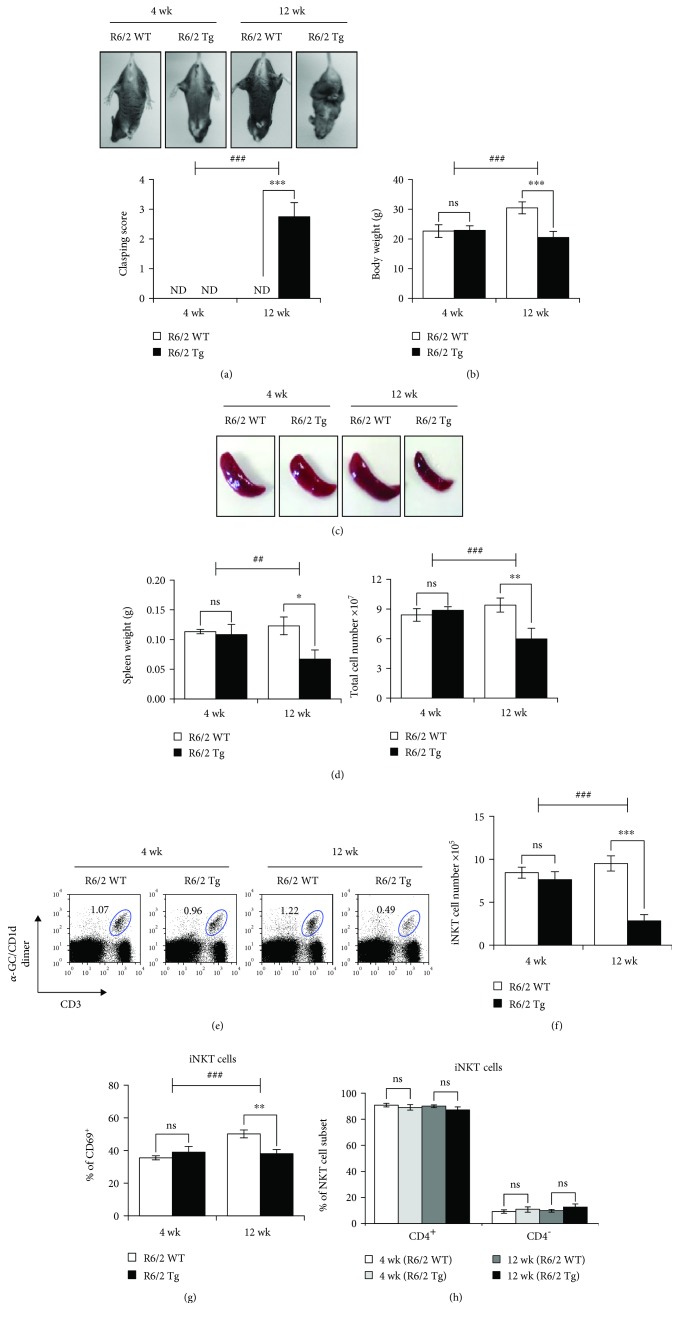
HD onset in R6/2 Tg mice is associated with reduced levels of iNKT cells. (a) Both clasping test and (b) body weight were evaluated from WT littermates and R6/2 Tg mice at 4 or 12 weeks of age. (c-d) Spleens were prepared from R6/2 WT littermates or R6/2 Tg mice at 4 or 12 weeks of age, and the spleen weight and splenocyte number were evaluated. (e) The frequency, (f) cell number, (g) surface CD69 expression, and (h) subsets in splenic iNKT cells (*α*-GalCer/CD1d-dimer^+^CD3^+^) were determined by flow cytometry. The mean values ± SD (*n* = 4; *n* indicates the number of mice per group in the experiment; Student's *t*-test; ^∗^*P* < 0.05, ^∗∗^*P* < 0.01, and ^∗∗∗^*P* < 0.001) are shown (ns: not significant). Two-way ANOVA (genotype × time) showed an interaction between these two factors (^##^*P* < 0.01 and ^###^*P* < 0.001).

The R6/2 Tg mouse is the best-characterized and most widely used animal model for HD, which represents a behavioral, progressive neurological phenotype similar to HD patients [2]. To confirm the characteristics during natural disease progression in R6/2 Tg mice, we compared the disease phenotypes including the clasping behavior and body weight of R6/2 Tg mice with those of WT littermate controls at both 4 and 12 weeks of age. Twelve-week-old but not 4-week-old R6/2 Tg mice exhibited significantly higher levels of clasping phenotype (Figure 1(a)) and displayed reduced body weight compared with WT littermates of the same age (Figure 1(b)).

Since altered immune responses have been implicated in the pathogenesis of HD [15], we compared the spleen weight and total cell number in R6/2 Tg mice at both 4 and 12 weeks of age. Unlike 4-week-old R6/2 Tg mice, both the spleen weight and total splenocytes were significantly decreased in 12-week-old R6/2 Tg mice compared with those of their WT littermates (Figures 1(c) and 1(d)). These data indicate that development of HD is strongly associated with a reduction in immune cells.

In previous reports, iNKT cells were shown to display immune modulating effects in peripheral lymphoid organs such as the spleen [16, 17]. Thus, we explored changes in splenic iNKT cells during disease progression in R6/2 Tg mice. We found that iNKT cells were dramatically reduced in 12-week-old R6/2 Tg mice compared to 4-week-old control groups, suggesting that iNKT cells might be involved in HD pathogenesis of R6/2 Tg mice (Figures 1(d)–1(f)). Next, to examine the activation status of iNKT cells, we compared CD69 surface expression between R6/2 Tg and WT littermate control mice. The frequency of CD69^+^ iNKT cells was decreased in R6/2 Tg mice with HD (Figure 1(g)). Since iNKT cells can be classified into two populations (CD4^+^ and CD4^−^) according to CD4 expression [18], we examined which iNKT cell subset was affected in R6/2 Tg mice. However, no biased reduction in iNKT cell subsets was observed (Figure 1(h)). Taken together, our data indicate that total iNKT cell numbers are reduced during HD progression in R6/2 Tg mice.

### 3.2. Absence of iNKT Cells Does Not Alter the Development of HD in R6/2 Tg Mice

Recently, we demonstrated that repeated treatment with low-dose LPS could attenuate HD progression in R6/2 Tg mice through modulation of innate immune cells such as dendritic cells (DCs) and macrophages [19]. Since iNKT cells are one of the innate-like T cells that play important roles in initiating immune responses, and because we had observed iNKT cell alterations during HD disease progression, we speculated that deficiency of iNKT cells in R6/2 mice might alter the progression of HD.

**Figure 2:**
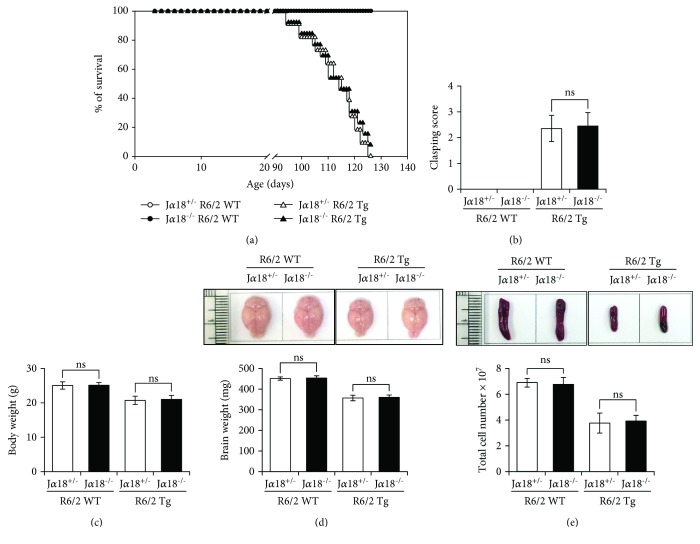
The absence of iNKT cells does not alter the development of HD in R6/2 Tg mice. (a) The survival rates of J*α*18^+/−^ R6/2 WT, J*α*18^−/−^ R6/2 WT, J*α*18^+/−^ R6/2 Tg, and J*α*18^−/−^ R6/2 Tg mice were monitored every week starting from 5 weeks of age for a total of 14 weeks. (b-c) These mice were also tested for hindlimb clasping behavior and weighed at 12 weeks of age. (d) Brains were harvested from these mice at 12 weeks of age, and their brain weights were evaluated. (e) Spleens were prepared from these mice at 12 weeks of age, and the total number of their splenocytes was evaluated. The mean values ± SD (*n* = 5-13 in a, b, and c; *n* = 4 in d and e; *n* indicates the number of mice per group in the experiment) are shown (ns: not significant).

To address this question, we generated iNKT cell-deficient R6/2 Tg mice by crossing R6/2 Tg mice with J*α*18 KO (J*α*18^−/−^) mice. Then, we compared the pathological severity of HD between J*α*18^−/−^ R6/2 Tg and J*α*18^+/−^ R6/2 Tg mice by measuring several clinical parameters including survival, clasping score, and body weight. Unexpectedly, our results showed that survival rates were not significantly changed in J*α*18^−/−^ R6/2 Tg mice (lacking iNKT cells) compared with those in J*α*18^+/−^ R6/2 Tg littermates (Figure 2(a)). Furthermore, neither clasping score nor body weight were significantly affected by the absence of iNKT cells (Figures 2(b) and 2(c)).

A previous study showed that the whole-brain volumes of HD patients were significantly reduced by about 25% in advanced HD [20]. Consistent with this finding, our results showed that the total brain weight was reduced by 21% in 12-week-old R6/2 Tg mice compared with WT littermates, but deficiency of iNKT cells did not affect this parameter (Figure 2(d)). Moreover, the lack of iNKT cells had little impact on spleen size (Figure 2(e)). These results indicate that unactivated iNKT cells are not major contributors to the progression of symptoms in R6/2 Tg mice.

### 3.3. Repeated *In Vivo* Activation of iNKT Cells with *α*-GalCer Enhances the Development of HD in R6/2 Tg Mice

**Figure 3:**
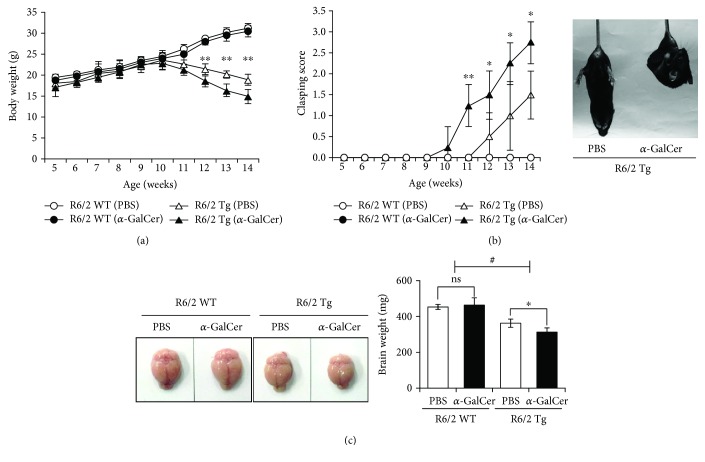
Repeated activation of iNKT cells with *α*-GalCer enhances the development of HD in R6/2 Tg mice. (a-c) Both R6/2 WT and Tg mice were i.p. injected with either PBS (*n* = 4) or *α*-GalCer (2 *μ*g) (*n* = 4) once per week from 5 weeks of age for a total of 9 weeks. (a-b) These mice were also weighed and tested for hindlimb clasping behavior weekly starting from 5 weeks of age for a total of 9 weeks. (c) Brains were harvested from these mice at 9 weeks after *α*-GalCer injection, and their brain weights were evaluated. The mean values ± SD (*n* = 4, ^∗^*P* < 0.05 and ^∗∗^*P* < 0.01) are shown (ns: not significant). Two-way ANOVA (genotype × treatment) showed an interaction between these two factors (^#^*P* < 0.05).

HD patients might be exposed to both endogenous and exogenous glycolipid antigens capable of activating iNKT cells [21–23]. Thus, we wanted to determine whether activation of iNKT cells can impact the pathogenesis of HD in R6/2 Tg mice. To assess this possibility, we took advantage of *α*-GalCer which is a well-known iNKT cell agonist. After repeated *in vivo* activation of iNKT cells with *α*-GalCer, R6/2 Tg mice were monitored to measure the clinical parameters including body weight, clasping score, and brain weight. We found that repeated *α*-GalCer injection significantly reduced body weight in R6/2 Tg mice compared with PBS-injected control mice (Figure 3(a)). In addition, *α*-GalCer-injected R6/2 Tg mice displayed clasping symptoms much earlier than PBS-injected R6/2 Tg mice (Figure 3(b)). Moreover, *α*-GalCer injection caused severe reduction of the brain weight in R6/2 Tg mice compared with PBS-injected controls (Figure 3(c)). These findings strongly suggest that iNKT cells can facilitate the initiation of HD clinical phenotypes in R6/2 Tg mice upon *α*-GalCer stimulation.

### 3.4. Exacerbation of HD Pathogenesis by *α*-GalCer Treatment Correlates with Increased Infiltration of iNKT Cells into the Brain

**Figure 4:**
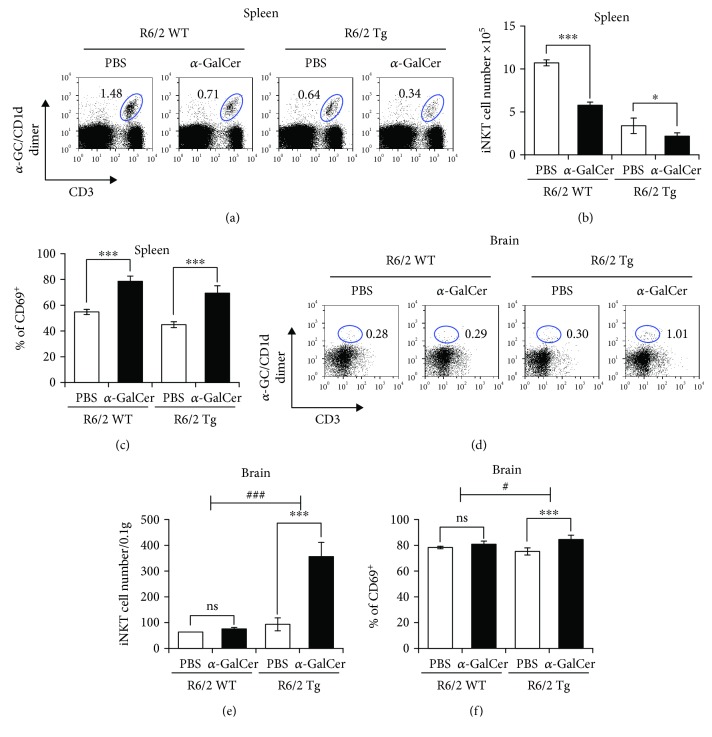
Exacerbated HD pathogenesis induced by *α*-GalCer treatment is associated with increased infiltration of iNKT cells into the brain. (a-f) Both R6/2 WT and Tg mice were i.p. injected with either PBS (*n* = 4) or *α*-GalCer (2 *μ*g) (*n* = 4) once per week starting from 5 weeks of age for a total of 9 weeks. (a-c) Splenocytes were prepared from these mice at 9 weeks after *α*-GalCer injection. (a) The frequency, (b) cell number, and (c) activation marker (CD69) expression in splenic iNKT cells (*α*-GalCer/CD1d-dimer^+^CD3^+^) from these mice were determined by flow cytometry. The mean values ± SD (*n* = 4, ^∗^*P* < 0.05 and ^∗∗∗^*P* < 0.001) are shown. (d-f) Brains were harvested from these mice at 9 weeks after *α*-GalCer injection, and the brain MNCs were prepared using a Percoll gradient. (d-e) The absolute numbers of brain iNKT cells (*α*-GalCer/CD1d-dimer^+^CD3^+^) per 0.1 gram were determined by flow cytometry. The mean values ± SD (*n* = 4, ^∗∗∗^*P* < 0.001) are shown (ns: not significant). (f) Surface CD69 expression in splenic and brain iNKT cells (*α*-GalCer/CD1d-dimer^+^CD3^+^) was determined by flow cytometry. The mean values ± SD (*n* = 4, ^∗∗^*P* < 0.01) are shown (ns: not significant). Two-way ANOVA (genotype × treatment) showed an interaction between these two factors (^#^*P* < 0.05 and ^###^*P* < 0.001).

We next examined the effect of repeated *α*-GalCer treatment on the splenic iNKT cell population in R6/2 Tg mice. Both the frequency (Figure 4(a)) and absolute cell number (Figure 4(b)) of splenic iNKT cells among total splenocytes were significantly decreased in *α*-GalCer-treated R6/2 Tg mice compared with the PBS-treated littermate control group (Figure 4(a)). To test whether *α*-GalCer treatment induces the activation of splenic iNKT cells, we measured surface expression levels of CD69, an early marker of iNKT cell activation. Our results demonstrated that splenic iNKT cells from *α*-GalCer-injected R6/2 Tg mice displayed significantly increased expression of CD69 compared with the PBS-injected littermate control group (Figure 4(b)), indicating that repeated *α*-GalCer treatment induced activation rather than anergy of iNKT cells. Thus, our data suggest that repeated *α*-GalCer activation induces activation-induced cell death (AICD) of iNKT cells, consequently resulting in a numerical reduction of iNKT cells [24].

It has been reported that *in vivo* injection of *α*-GalCer induces the infiltration of iNKT cells into the brain and spinal cord during EAE, ultimately resulting in inhibition of EAE [25]. To examine whether repeated *α*-GalCer treatment can trigger iNKT cell infiltration into the brain during HD, we measured the frequency of iNKT cells in the brain in both R6/2 WT and Tg mice after repeated *α*-GalCer treatment. The frequency and absolute cell number of the brain-infiltrating iNKT cells in *α*-GalCer-injected R6/2 Tg mice were 3- to 4-fold higher compared with PBS-injected R6/2 Tg mice, whereas *α*-GalCer injection did not affect the infiltration of iNKT cells into the brain of WT littermates.

Since T cells in the brain express high levels of CD69 [26], we next examined CD69 expression on iNKT cells in the brain of R6/2 Tg mice. Compared with splenic iNKT cells, brain-resident iNKT cells expressed higher levels of CD69 in both R6/2 WT and R6/2 Tg mice, even in the absence of *α*-GalCer treatment. Further, *α*-GalCer slightly but significantly increased CD69 expression on brain iNKT cells in R6/2 Tg but not WT littermate control mice. Taken together, these findings indicate that the brain infiltration of iNKT cells upon *α*-GalCer stimulation might be associated with the acceleration of HD symptoms in R6/2 Tg mice.

## 4. Discussion

Although mutant HTT is expressed in the immune system as well as the brain [27], the role of the immune response in the pathogenesis of HD is controversial [28–30]. Here we investigated the role of iNKT cells in the development of HD in R6/2 Tg mice and whether repeated iNKT cell activation with *α*-GalCer could influence HD progression. We found that during steady-state conditions, iNKT cells have little impact on HD pathogenesis. However, repeated stimulation of iNKT cells with *α*-GalCer exacerbated the pathogenesis of HD in R6/2 Tg mice. Moreover, repeated *α*-GalCer-mediated inflammation enhanced iNKT cell infiltration into the brain, which might be involved in the rapid onset of HD development in R6/2 Tg mice.

Recently, we have demonstrated that repeated injection of low-dose LPS attenuated the progression of HD in R6/2 Tg mice, suggesting that low levels of inflammation have a beneficial effect by delaying the progression of HD [19]. Previous studies have shown that LPS-activated microglia exhibit increased GLT-1 expression and glutamate uptake capacity in a TNF*α*-dependent manner [31], suggesting that microglia-mediated changes in glutamate uptake might be associated with clinical symptoms of HD. Additionally, microglia from premanifest human HD patients showed impaired migratory capacity due to the accumulation of mutant HTT [8].

It has been reported that *α*-GalCer stimulation can induce iNKT cells to produce both anti-inflammatory cytokines (e.g., IL4, IL13, and IL10) and pro-inflammatory cytokines (e.g., IFN*γ*, TNF*α*, and IL17), ultimately determining the outcome of autoimmune diseases such as EAE [32]. For example, activation of iNKT cells attenuated EAE by inhibiting macrophage infiltration and microglia-mediated neuroinflammation [33]. However, iNKT cells can infiltrate the brain within 48 hr during cerebral ischemia, and *α*-GalCer treatment significantly increases these harmful effects [34]. In particular, repeated exposure of mice to *α*-GalCer promotes the development of IL10-producing iNKT cells (NKT10 cells) with anti-inflammatory abilities [35]. It will be of interest to test whether NKT10 cells are induced by the repeated injection of *α*-GalCer in our experimental setting. This subset of iNKT cells might inhibit the M1-like functions of microglia that are capable of clearing aggregated proteins during HD pathogenesis.

Unlike circulating central memory T cells expressing sphingosine-1-phosphate (S1P1), tissue-resident T cells express high levels of CD69, antagonizing S1P1, which is essential for tissue egress [36]. Moreover, the brain-resident T cells are characterized as CD69-positive and Bcl-2^hi^ cells [26]. Since we have shown that the brain-infiltrating iNKT cells following injection of *α*-GalCer are mostly CD69-positive cells in R6/2 Tg mice, our results strongly suggest that CD69^+^ iNKT cells might be closely correlated with HD progression.

Endogenous [22] and exogenous [21] glycolipid antigens can activate iNKT cells in a CD1d-dependent manner. Moreover, enterogenous glycolipids derived from commensal bacteria circulate throughout the body and can also be recognized by iNKT cells [23]. It has been reported that microbial metabolites play a critical role in shaping the development and function of host immune responses [37]. For instance, long-chain fatty acids such as palmitate inhibit iNKT cell-mediated inflammatory responses, thereby leading to suppression of arthritis [38]. Thus, it is interesting to examine whether regulation of mucosal immune responses by natural products or microbial metabolites with iNKT cell-modulating activities can be effective in controlling HD pathogenesis [39].

## 5. Conclusion

In this study, we demonstrated that resting iNKT cells do not influence HD pathogenesis, but iNKT cells activated by repeated injection of *α*-GalCer exacerbate HD progression in R6/2 Tg mice, identifying iNKT cells as novel targets to modulate HD pathology. Considering our recent study that Th1-type immune responses elicited by repeated low-dose LPS treatment ameliorate HD, we propose that disease exacerbation by repeated *α*-GalCer treatment might be related to Th2-deviated immune responses. Thus, it will be worthwhile to investigate whether HD pathogenesis can be altered by treating R6/2 Tg mice with Th1-cytokine biasing iNKT cell ligands such as *α*-C-Gal.

## Data Availability

The data used to support the findings of this study are available from the corresponding author upon request.
